# Caveolin-3 and Caveolin-1 Interaction Decreases Channel Dysfunction Due to Caveolin-3 Mutations

**DOI:** 10.3390/ijms25020980

**Published:** 2024-01-12

**Authors:** Patrizia Benzoni, Elisabetta Gazzerro, Chiara Fiorillo, Serena Baratto, Chiara Bartolucci, Stefano Severi, Raffaella Milanesi, Melania Lippi, Marianna Langione, Carmen Murano, Clarissa Meoni, Vera Popolizio, Alessandro Cospito, Mirko Baruscotti, Annalisa Bucchi, Andrea Barbuti

**Affiliations:** 1 The Cell Physiology MiLab, Department of Biosciences, Università degli Studi di Milano, 20133 Milan, Italy; 2 Unit of Muscle Research, Experimental and Clinical Research Center, Cooperation between the Max-Delbrück-Center for Molecular Medicine in the Helmholtz Association and Charité-University Berlin, 13125 Berlin, Germany; 3 Child Neuropsychiatry Unit, IRCCS Istituto Giannina Gaslini, DINOGMI-University of Genova, 16147 Genova, Italy; 4 Center of Translational and Experimental Myology, IRCCS Istituto Giannina Gaslini, 16147 Genova, Italy; 5 Department of Electrical, Electronic and Information Engineering “Guglielmo Marconi”, University of Bologna, 47521 Cesena, Italy

**Keywords:** caveolin-3, caveolin-1, electrophysiology, HCN4, Kv1.5, Kir2.1, caveolinopathies

## Abstract

Caveolae constitute membrane microdomains where receptors and ion channels functionally interact. Caveolin-3 (cav-3) is the key structural component of muscular caveolae. Mutations in *CAV3* lead to caveolinopathies, which result in both muscular dystrophies and cardiac diseases. In cardiomyocytes, cav-1 participates with cav-3 to form caveolae; skeletal myotubes and adult skeletal fibers do not express cav-1. In the heart, the absence of cardiac alterations in the majority of cases may depend on a conserved organization of caveolae thanks to the expression of cav-1. We decided to focus on three specific cav-3 mutations (Δ62-64YTT; T78K and W101C) found in heterozygosis in patients suffering from skeletal muscle disorders. We overexpressed both the WT and mutated cav-3 together with ion channels interacting with and modulated by cav-3. Patch-clamp analysis conducted in caveolin-free cells (MEF-KO), revealed that the T78K mutant is dominant negative, causing its intracellular retention together with cav-3 WT, and inducing a significant reduction in current densities of all three ion channels tested. The other cav-3 mutations did not cause significant alterations. Mathematical modelling of the effects of cav-3 T78K would impair repolarization to levels incompatible with life. For this reason, we decided to compare the effects of this mutation in other cell lines that endogenously express cav-1 (MEF-STO and CHO cells) and to modulate cav-1 expression with an shRNA approach. In these systems, the membrane localization of cav-3 T78K was rescued in the presence of cav-1, and the current densities of hHCN4, hKv1.5 and hKir2.1 were also rescued. These results constitute the first evidence of a compensatory role of cav-1 in the heart, justifying the reduced susceptibility of this organ to caveolinopathies.

## 1. Introduction

Caveolins are structural membrane proteins involved in the formation and stabilization of caveolae [1,2]. These specialized lipids rafts are cup-shaped invaginations found in cholesterol- and sphingolipid-rich regions of the plasma membrane of most mammalian cells, among which are skeletal fibers and cardiac cells. Many receptors, enzymes and ion channels have been found to directly interact with caveolins and/or localize to caveolae, which constitute micro-domains where proteins are clustered, favoring their functional interactions [3] and/or their modulation [4]. This makes caveolae of primary importance for many cellular functions, including endocytosis, lipid homeostasis and intracellular signaling [5]. Moreover, caveolae contribute to the protection of the plasma membrane from mechanical stress, and to initiate its repair [6,7]. 

In humans, caveolin proteins are encoded by three genes: caveolin-1 (*CAV1*), caveolin-2 (*CAV2*) and caveolin-3 (*CAV3*). *CAV1* and *CAV2*, both found on chromosome 7, are generally co-expressed in all cell types that show caveolae, while *CAV3*, located on chromosome 3, is the muscle-specific isoform present in skeletal, cardiac and smooth muscle cells [8]. 

Cav-1 forms large homo-oligomers and forms caveolae in vivo and can also form hetero-oligomers with cav-2 and cav-3 in cardiomyocytes. In skeletal muscles, *CAV1* is expressed only at the stage of satellite cells and myoblasts and it is downregulated in myotubes and adult fibers [9].

The knockout of *CAV1* and/or *CAV3* in mice causes the complete disappearance of caveolae from the tissues where they are normally expressed [10]. 

Mutations in the *CAV3* gene can give rise to a family of neuromuscular disorders called caveolinopathies. Such disorders mostly impact the skeletal muscle and are characterized by alterations in the morphology of fibers, myalgia, increased creatine kinase (CK) levels and uncontrolled contractile events [11]. So far, more than 40 pathogenic mutations have been identified in humans [12]. *CAV3* mutations have been found all over the cav-3 protein, though most of them are located at the N-terminus and in the oligomerization and intramembrane domains. Typically, the mutations cause decreased membrane expression and/or retention of cav-3 in the perinuclear region/Golgi. Although the mechanisms by which *CAV3* mutations contribute to pathogenesis are different, the pathological phenotypes which they are mostly associated with are limb-girdle muscular dystrophy 1c (LGMD-1c), rippling muscle disease (RMD), hyperCKemia (HCK), distal myopathy (DM) and myalgia [13]. 

*CAV3* is also expressed in the cardiac muscle and, even if rare, cases of associations between mutations in its sequence with cardiac diseases have been reported, including long QT syndrome (LQTS), atrial fibrillation (AF), sudden infant death syndrome (SIDS) and hypertrophic cardiomyopathy (HCM) [4,13,14,15,16]. 

Several ion channels localize to caveolae; thus, reduction in caveolae abundance can also be responsible for alterations in ion channel trafficking/modulation in cardiac cells [17]. However, the majority of *CAV3* mutations do not cause cardiac phenotypes or their onset is typically delayed compared to that in skeletal muscle [1]. This evidence suggests the presence of compensatory/protective mechanisms to cav-3 alterations in the heart. 

In this work, we investigated the possible arrhythmogenic effects of three *CAV3* heterozygous mutations found in patients with neuromuscular diseases: p.Δ62-64YTT; p. T78K; and p.W101C, located in different functional domains of the protein. We analyzed the electrical properties of different human cardiac ion channels, known to directly interact with cav-3: hHCN4 [3], hKv1.5 [18] and hKir2.1 [19], by co-transfecting them with both WT and mutated cav-3 in various cellular systems either nonexpressing caveolins (cav-1 free MEF) or expressing various levels of cav-1 (STO-MEF, Shcav-1 STO and CHO cells). 

## 2. Results

To date, several mutations in the *CAV3* gene are known; these are associated with either muscle or heart pathologies. The knockout or knockdown of *CAV3* in different heart models has been demonstrated to be functionally harmful for cardiomyocytes [1,4,14]. Nevertheless, well-known protein mutations of cav-3 associated with dystrophies (e.g., T78K; ∆TFT; W101C) characterized by the complete absence of caveolin-3 in the plasma membrane of muscle biopsies, do not give any cardiac alteration. On which basis this happens is still not entirely clear since cav-3 is expressed both in muscle fibers and cardiomyocytes. 

**Figure 1:**
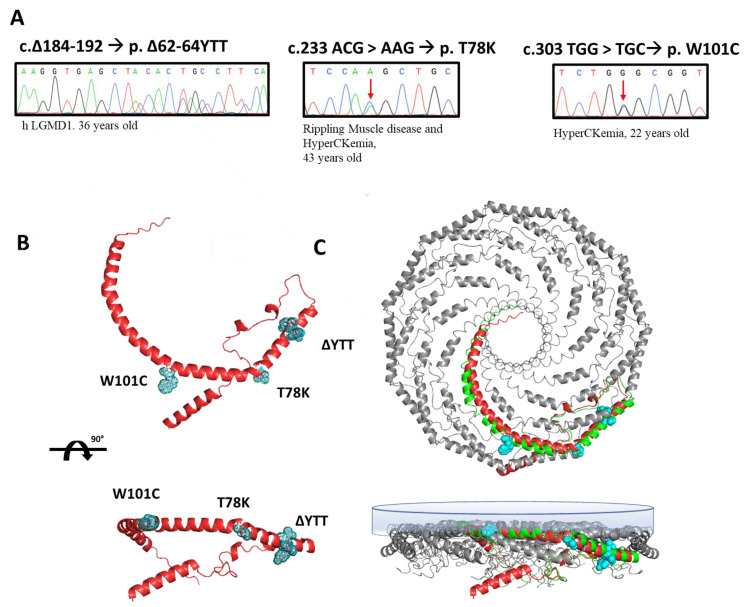
(**A**) Sanger sequencing of the regions of the DNA of patients containing the mutations in the *CAV3* gene (red arrows indicate the mutated nucleotide). Next to each mutation the associated disease phenotype and the age of onset are reported. (**B**) Ninety-degree rotated views of the predicted secondary structure of human cav-3 alone (AF-P56539-F1-model_v1). The residues involved in the three mutations are highlighted in light blue. (**C**) Ninety-degree rotated views of secondary structure of 11 human cav-1 complexes (panel bottom left, mmdb_7SC0). In the bottom-right panel, the single monomer of cav-1 (green) is aligned to the predicted structure of human cav-3 (red) in the multimeric Cav complex (AF-P56539-F1-model_v1). The inner membrane is depicted as the blue surface.

In this paper, we selected three heterozygous mutations found in three patients followed at Gaslini Hospital in Genoa. Specifically, the sequencing analyses of the *CAV3* gene identified, respectively: (i) a deletion of nine base pairs (184–192) corresponding to a deletion of p.∆62-64 YTT residues within the cav-3 scaffolding domain (CSD), in a patient affected by limb-girdle muscular dystrophy (LGMD-1c); (ii) the substitution of threonine 78 with a lysine (p.T78K), a conserved residue at the border region between the CSD and intra-membrane domain, in a patient affected by rippling muscle disease (RMD); (iii) the substitution of tryptophan 101 with a cysteine (p.W101C), a conserved residue within the intra-membrane domain in a patient affected by asymptomatic hyperCKemia. The DNA sequencing of *CAV3* mutants, the disease phenotype and age of onset are reported in Figure 1A.

We reported the relative position of these mutations in the predicted secondary structure of cav-3 (red structure, Figure 1B), in which we highlighted the mutated residues (light blue). In Figure 1C, the cav-3 structure is overlapped and aligned on the recently published secondary structure obtained by CryoEM of cav-1 oligomers, in green (modified from [20]). Although not sustained by real structural data, this image gives a hint on a possible organization of cav-1 (green)/cav-3 (red) oligomers. It is interesting to note the location of the mutations analyzed in this work, within the caveolin complex: the YTT sequence is in the cytoplasmic region within the CSD, open to interactions; the T78 residue is instead at the bending point of the structure at the interface with the membrane (close to the CSD); while the W101 is embedded in the cytosolic leaflet of the membrane.

**Figure 2:**
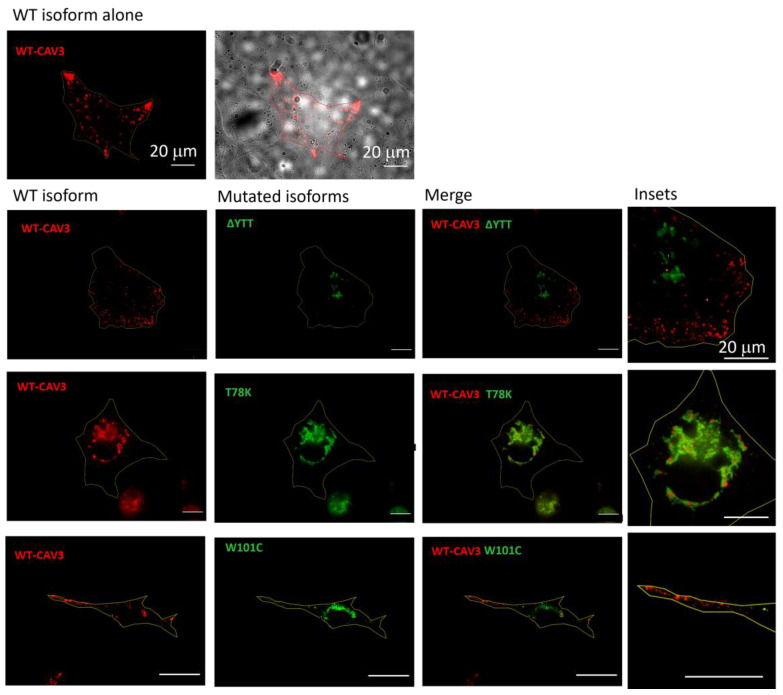
Representative fluorescence images of MEF-KO cells transfected with either the WT cav-3-DsRed (red, left column) or together with the mutated EGFP-cav-3, as indicated (green, middle column) (*n* = 3). Right panels show both signals overlapped (merge) and the insets (far right) show magnification of the signals to appreciate the detailed distribution of the two caveolin isoforms. Yellow line describes the cell profile captured in brightfield, as shown for the WT in the top panels. Scale bar = 20 µm.

Using murine fibroblasts KO for cav-1 (MEF-KO), we first investigated whether these three mutations alter the localization of caveolin proteins, considering the heterozygous condition of patients. Using this model, we intended to replicate a situation similar to that in skeletal muscle in which cav-1 is completely absent in adult fibers [21]. We transfected the different isoforms (WT and mutated) of cav-3 linked to either DsRed (WT) or EGFP (mutated), respectively. After 40 h from transfection, the relative fluorescence was analyzed. In Figure 2, representative images of transfected cells for each heterozygous mutation are reported. All the mutated forms are localized inside the cell close to the nucleus, while the localization of the WT isoform is close to the plasma membrane (see also the merge of the brightfield image and WT cav-3 localization in the top center image). Cav-3 is strongly mislocalized only in the presence of the T78K mutation, where the red signal is completely colocalized with the mutated (green) isoform inside the cell. In the presence of both ∆YTT and W101C mutations, the WT (red) signal seems to reach the cell periphery. However, it is impossible to quantify how much WT protein is expressed compared to the total transfected and whether a similar situation could also occur at the skeletal-muscle level in the patient, as the physiological expression of the proteins may differ.

On the other hand, these data clearly indicate a negative dominance of the T78K mutation, and this could lead to a greater effect of this mutation on cellular function. Confirming the findings in the MEF-KO model, immunohistochemistry on muscle biopsies of the patient with the CAV3 T78K mutation shows an almost absence of cav-3 signal at the sarcolemma (Supplementary Figure S1).

In order to better understand the potential cardiac implications of cav-3 mutations linked to dystrophic phenotypes, we transfected the MEF-KO cells with cardiac ion channels (hHCN4, hKv1.5, and hKir2.1) known to functionally interact with cav-3. We then compared their properties by co-expressing either the cav-3 WT alone or the WT plus the mutated cav-3 construct, mimicking the heterozygous condition of the patient. We chose not to transfect the channels for calcium and sodium currents since their changes would hardly ever result in a normal ECG.

**Figure 3:**
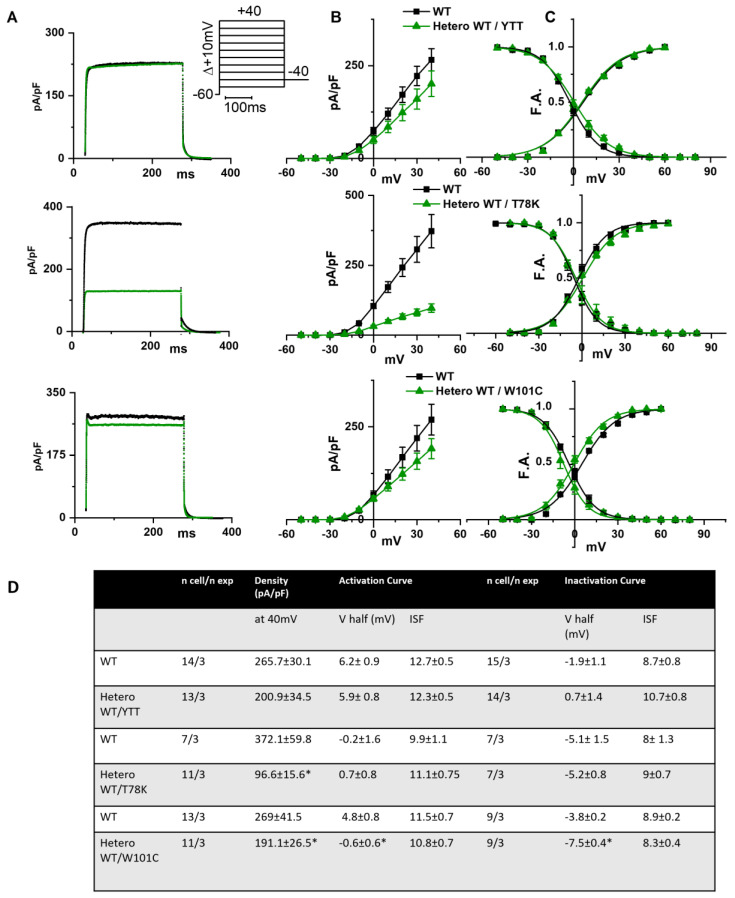
(**A**) Representative current traces recorded during activation voltage protocol, is reported in the inset, from MEF-KO cells co-transfected with hKv1.5 and either homozygous WT cav-3 (black line) or heterozygous WT/mutated cav-3 (green line). (**B**) Mean current density–voltage relations in WT condition (black squares) vs. WT/mutated cav-3 heterozygous condition (green triangles). (**C**) Mean activation and inactivation curves fitted to the Boltzmann equation (symbols and colors as in (**B**)). The top to bottom cav-3 variants examined consist of ∆YTT, T78K, and W101C. (**D**) Summary table of mean data ± SEM. * *p* < 0.05 by Student’s *t*-test.

In the left of Figure 3A the following are shown: hKv1.5 current traces at 40 mV recorded from MEF-KO cells transfected with only WT cav-3 (black line) or with the ∆YTT cav-3 mutation (top panel, green line); cav-3 T78K mutation (middle panels, green line); and W101C mutation (bottom panels, green line). The ∆YTT heterozygous mutation affected neither the IKv1.5 current density (Figure 3B, center) nor the activation and inactivation curves (Figure 3C, right). The presence of both the T78K and W101C decreased the peak current density at 40 mV of 74% and 29%, respectively (Figure 3B, top and bottom, respectively). The plot of the mean activation/inactivation curves (Figure 3C, bottom) shows that hKv1.5 activated at significantly more negative voltages when expressed with the W101C (green triangle) than with the WT cav-3 alone (black square); the leftward shifts were 5.4 mV in AC and 3.7 mV in IC. 

**Figure 4:**
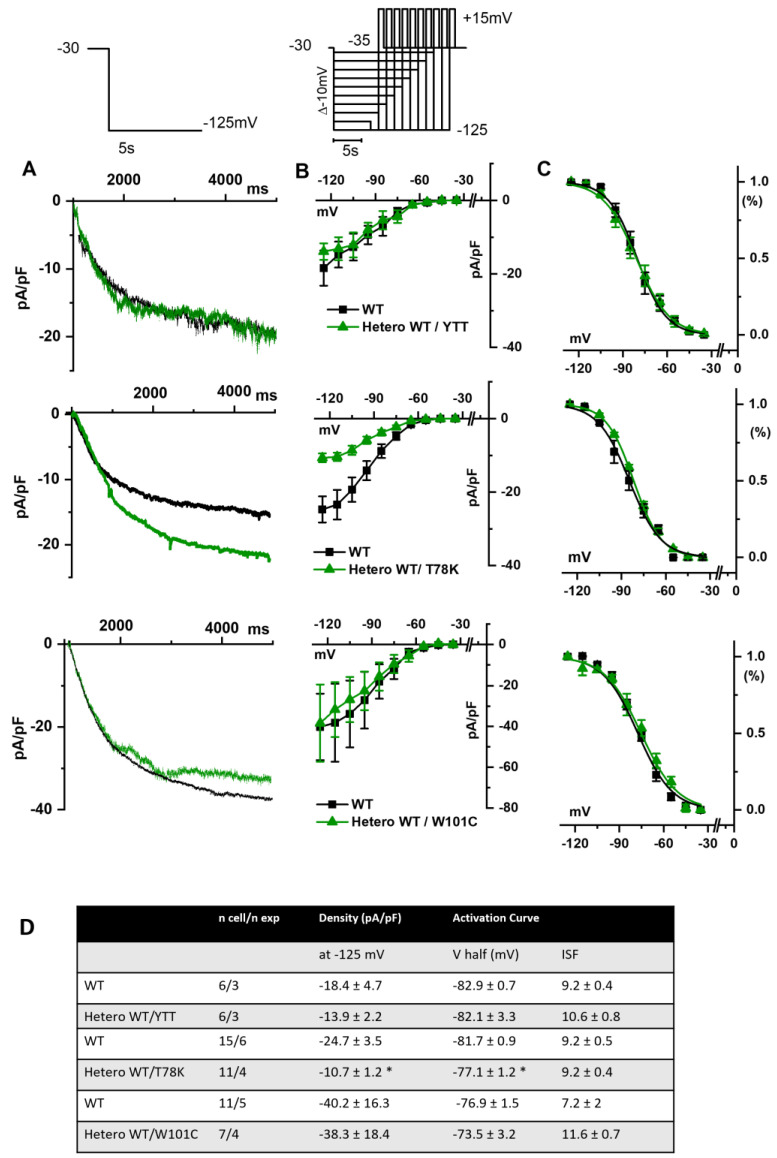
(**A**) Representative current traces recorded during the activation voltage step at −125 mV as reported in the top panel, from MEF-KO cells co-transfected with hHCN4 and either homozygous WT cav-3 (black line) or heterozygous WT/mutated cav-3 (green line). (**B**) Mean current density–voltage relations in WT condition (black squares) vs. WT/mutated cav-3 condition (green triangles) recorded during activation voltage protocol as reported in the top panel (**C**) Mean activation curves fitted to the Boltzmann equation (symbols and colors as in (**B**)). The top-to-bottom cav-3 variants examined consist of ∆YTT, T78K, and W101C. (**D**) Summary table of mean data ± SEM. * *p* < 0.05 by Student’s *t*-test.

In Figure 4A, hHCN4 current traces recorded at −125 mV from MEF-KO cells transfected with either the WT cav-3 (black line) or mutations in the heterozygous condition (green line) are shown. It is evident that only the T78K heterozygous mutation (middle Figure 4A,B) has a strong effect on this current. The hHCN4 mean current density reported in Figure 4B was similar to that of cells expressing only the WT or the heterozygous ∆YTT and W101C (top and bottom panels, respectively) over the whole range of voltages tested; when T78K cav-3 was expressed in heterozygosis, the mean hHCN4 current density significantly decreased by more than 56% (loss of function) (middle Figure 4B). Moreover, the plot of the mean activation curves shows that the presence of the T78K cav-3 induced a significant 4.6 mV positive shift in the voltage dependence (V_1/2_) of hHCN4 (gain of function).

**Figure 5:**
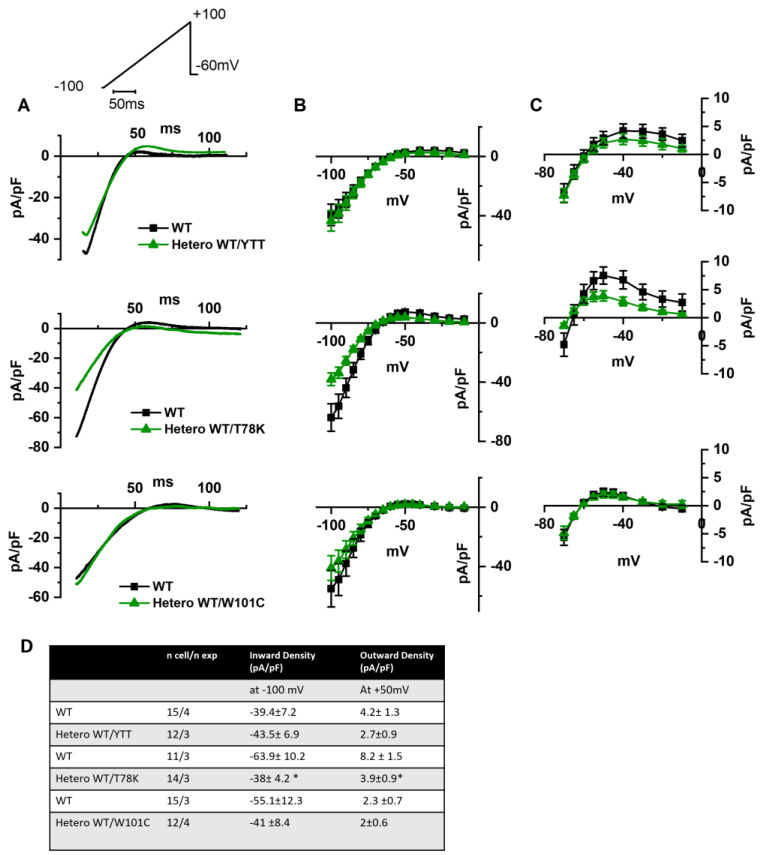
(**A**) Representative current traces recorded during the ramp protocol reported in the top panel, from MEF-KO cells co-transfected with hKir2.1 and either homozygous WT cav-3 (black line) or heterozygous WT/mutated cav-3 (green line) during a ramp voltage protocol from −100 to −20 mV. (**B**) Mean current density–voltage relations in WT (black squares) vs. WT/mutated cav-3 conditions (green triangles). (**C**) Right panels show specifically the outward component (from −70 to −10 mV; symbols and colors as in Figure 4B). The top to bottom cav-3 variants examined consist of ∆YTT, T78K, and W101C. (**D**) Summary table of mean data ± SEM. * *p*  <  0.05 by Student’s *t*-test.

In Figure 5A we report the representative traces of hKir2.1 current obtained by applying a depolarizing ramp protocol from −100 mV to −20 mV in MEF-KO cells transfected with either WT alone or with the various mutations in heterozygous conditions. In this case also, only the T78K co-expression significantly decreased the hKir2.1 current density both of the inward (−40.6%) and outward (−52.5%, Figure 5C) components. 

**Figure 6:**
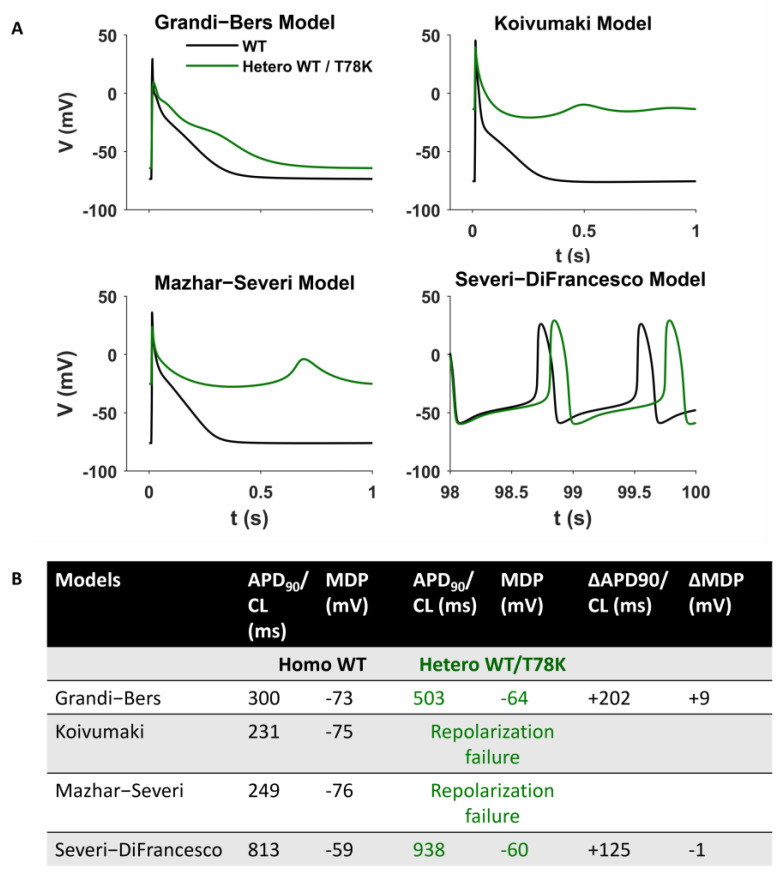
Computational Modelling of the T78K effect. (**A**) Mathematical models of both atrial and sinoatrial cells showed an arrhythmia. Representative action potentials generated using the Courtmanche, Koivumaki and Grandi–Bers human atrial cell models and the Severi–DiFrancesco rabbit sinoatrial cell model, respectively [22,23,24,25]. Black line, basal conditions (WT); green line, after insertion of the T78K cav-3-dependent alterations. (**B**) Summary table of the T78K effects on different mathematical models analyzed.

Since the above data point to a possible strong cardiac effect of the T78K variant, we adopted an in silico approach to verify the effects of this variant by numerical modelling (Figure 6). We introduced the I_f_, I_Kur_ and IK1 changes described above in three different models of human atrial cardiomyocytes (the only cardiomyocytes expressing I_Kur_) and in a model of pacemaker cardiomyocytes [22,23,24,25]. The presence of the T78K mutation induces changes in action potential that are not compatible with a physiological cardiac activity (such as failure of repolarization for two of the three atrial models) that does not correlate with the patient’s condition. 

We hypothesized that the CAV3 mutations like the T78K have a smaller impact on the cardiac muscle because of the co-expression of cav-1 in the heart, which can have a compensatory effect for this particular mutation, supplying cardiomyocytes with enough functional caveolae to rescue channel mislocalization and functional properties. Since the adult skeletal muscles do not express cav-1, this may be the reason for the higher sensitivity of this tissue and a more severe phenotype. 

To prove our hypothesis, we analyzed the electrical properties of those same ion channels co-transfected with the WT and the T78K cav-3 but in murine embryonic fibroblasts endogenously expressing cav-1 (STO-MEF and in CHO cells, see Supplementary Figure S2A, left). 

**Figure 7:**
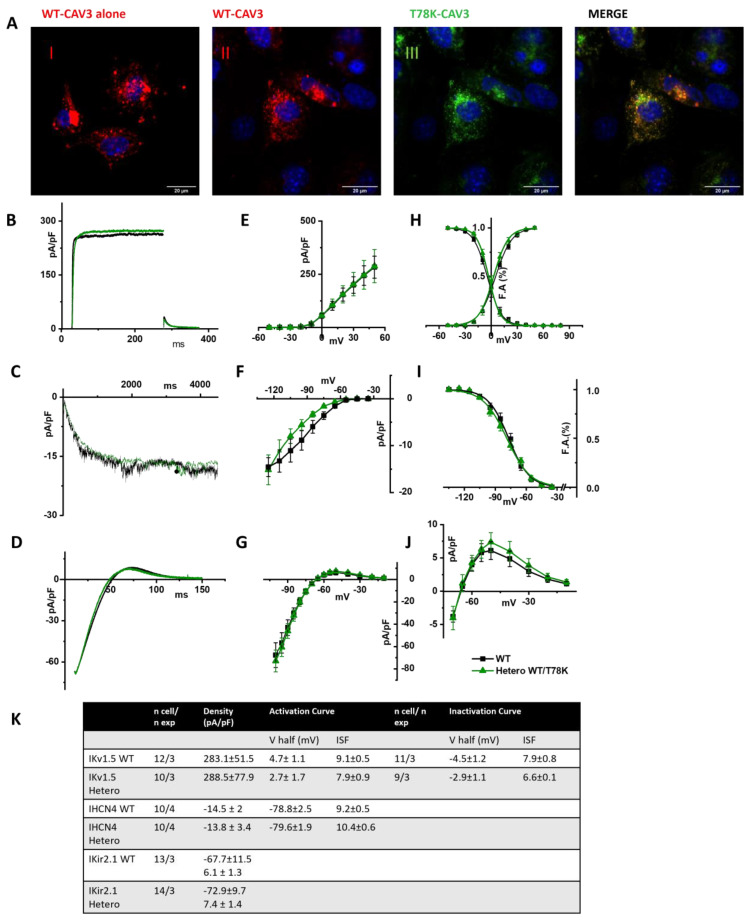
(**A**) Representative confocal images of STO-MEF cells transfected with either the WT-DsRed cav-3 (red, left panel) or together with the T78K cav-3 EGFP, as indicated (green) (*n* = 3). Far right panels show both signals overlapped (merge). Scale bar = 20 µm. (**B**–**D**) Representative current traces recorded from MEF-STO cells co-transfected with hKv1.5 (**B**), or hHCN4 (**C**) or hKir2.1 (**D**), and cav-3 in WT conditions is reported with black line, and WT/T78K condition as green line. (**E**–**G**) Mean current density–voltage relations recorded during specific activation voltage protocols from MEF-STO cells co-transfected with hKv1.5 (**E**); hHCN4 (**F**) or hKir2.1 (**G**) and cav-3 in WT vs. heterozygous conditions (symbols and colors as in panel (**B**)). (**H**) Mean activation and inactivation curves of Kv1.5 fitted to the Boltzmann equation (symbols as in panel (**E**)). (**I**) Mean activation curves of hHCN4 (symbols as in panel (**F**)) fitted to the Boltzmann equation. (**J**) Mean current density–voltage relation recorded during a ramp protocol displaying the outward Kir2.1 current (symbols as in panel (**G**)). (**K**) Summary table of mean data ± SEM.

In Figure 7A, representative images of STO-MEF cells transfected with either WT cav-3 alone (red, panel I) or WT cav-3 (red, panel II) together with T78K (green, panel III). The rightmost panels display the merged signals shown in II and III. The panels in Figure 7B–D show representative current traces of hKv1.5, hHCN4 and hKir2.1 in cells expressing the WT cav-3 alone (black traces) and in heterozygous condition (green traces). For each current, mean densities (Figure 7E, Figure 7F and Figure 7G, respectively) and activation/inactivation curves are shown (Figure 7H–J). Although the effects of the T78K mutation were highly significant in the MEF-KO system (and in the mathematical models tested), in the STO-MEF system no differences were detectable either in current densities or in kinetic properties. 

The same results were also obtained in CHO cells, another cellular system which endogenously expresses cav-1 (see Supplementary Figure S2A, right and Figure S2B). 

As further proof of the role of cav-1 in rescuing the loss-of-function effects of the T78K-cav-3, we generated a stable STO-MEF line with downregulated levels of cav-1, obtained through shRNA interference (see Section 4). In this system we repeated the recordings of only the hIKv1.5 current. We chose to assess the channel that is more sensitive to the cav-3 mutation and has a greater current density out of the three that were examined. In fact, the cav-3 T78K mutation in the MEF KO line resulted in a 74.1% decrease in Kv1.5 current; neither hHCN4 nor Kir2.1 exhibited a comparable change.

**Figure 8:**
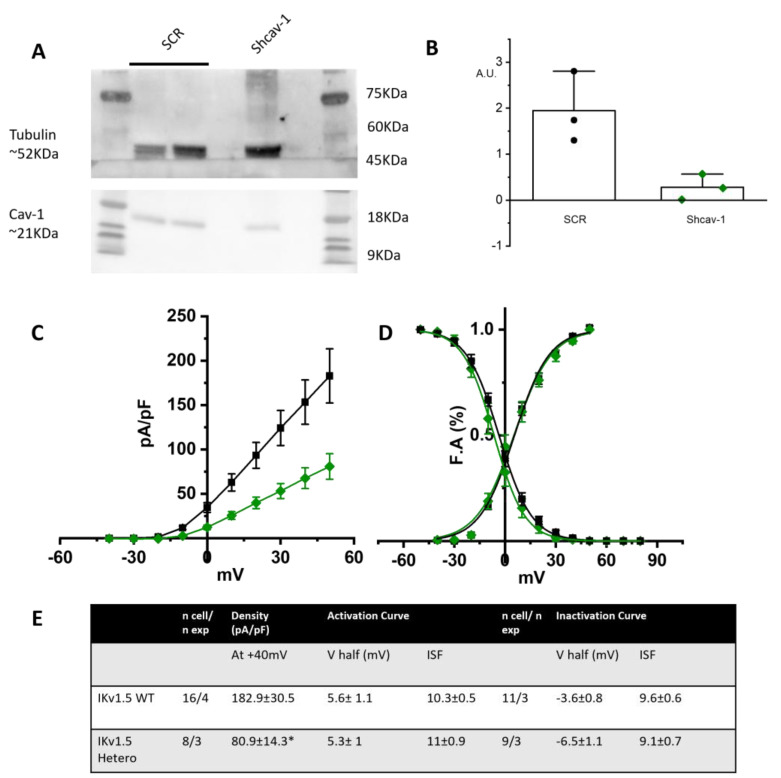
(**A**) Left, representative Western blot analysis for cav-1 expression in MEF stably transfected with pRS-Retroviral shRNA vector encoding scramble sequence (SCR) or shcav-1 sequence. Tubulin has been used as reference. (**B**) Plot of intensity blot analysis for cav-1 in three independent Western blots, normalized to tubulin. (**C**) Mean current density–voltage relations recorded during activation voltage protocol from MEF cells co-transfected with hKv1.5 and WT cav-3 (black squares) vs. heterozygous condition (green diamonds). (**D**) Mean activation and inactivation curves of Kv1.5 fitted to the Boltzmann equations (symbol as in (**C**)). (**E**) Summary table of mean data ± SEM. * *p* < 0.05 by Student’s *t*-test.

Figure 8A shows a representative Western blot and the quantification of cav-1 expression in the control line transfected with scramble sequence (SCR) and in the line transfected with shcav-1. We obtained a 78% reduction of cav-1 expression in the shcav-1 line (Figure 8A,B). 

STO-shcav1 were co-transfected with hKv1.5 and cav-3 in either WT homozygous or WT/T78K heterozygous conditions. In Figure 8C the mean density plot and the activation/inactivation curves are reported. The partial reduction in cav-1 was sufficient to induce a 55.6% reduction in the Kv1.5 current density in the heterozygous condition, while no changes were detectable in the kinetic properties (Figure 8D). 

## 3. Discussion

Caveolinopathies are a family of genetic disorders caused by mutations in the *CAV3* gene that lead to some rare forms of hereditary myopathies during childhood [26,27] and, more rarely, contribute to cardiac diseases such as familial hypertrophic cardiomyopathy (HCM), long-QT syndrome type 9 (LQT9), sudden infant death syndrome (SIDS) and atrial fibrillation [4,13,14]. Different phenotypes may be linked to the same mutation, but the co-existence of myopathy and cardiac disease has been rarely described, suggesting that the molecular networks supporting the role of cav-3 in skeletal muscle and heart may differ [28]. The heart is commonly involved in many dystrophies and other cardiac pathologies, such as, for example, hypertrophic cardiomyopathy (HCM) and dilated cardiomyopathy (DCM), conduction defects, and supraventricular and ventricular arrhythmias [13,29]. Nevertheless, the risk of cardiomyopathy linked to *CAV3* mutations remains poorly studied, and this assumes clinical relevance when considering improvement in the muscular therapy of young patients with caveolinopathies. This work aims to predict the risk of developing cardiac arrhythmias in patients with *CAV3* mutations located in different domains of the protein. We studied the following mutations: a deletion ∆YTT associated with limb-girdle muscular dystrophy; the point mutation T78K associated with rippling muscle disease [16]; and the point mutation W101C associated with hyperCKemia [30].

The pathological mechanisms of hereditary cardiac diseases due to *CAV3* mutations that have been described so far reported a direct influence of the CAV3 mutants on the activity of membrane ion channels localized within caveolae (Nav 1.5, Cav1.2, Kir2.1, Kv4.2, HCN4) [3,4,14,15,19,31,32]. 

Many *CAV3* mutations cause the retention of the protein in the Golgi’s network and/or in the perinuclear region, causing a strong reduction in or a complete absence of the protein at the sarcolemma [3]. This impaired trafficking of caveolins at the membrane may also affect the trafficking of interacting proteins such as ion channels, increasing the likelihood of developing arrhythmic events. For this reason, we initially wanted to investigate the localization and trafficking of the cav-3 mutants in the MEF-KO model, a cellular system not expressing cav-1 [3] and thus devoid of caveolae. 

It should be noted that, in the literature, the cellular line predominantly used to identify the arrhythmic effects of cav-3 mutations are HEK cells that either do not express or express barely detectable levels [19] of cav-1. Alternatively, KO mouse models were used [33]. 

Our data on the expression of the mutation ΔYTT show that its trafficking to the plasma membrane is impaired, and indeed it accumulates in the perinuclear region (likely in the Golgi). However, this behavior is independent from the trafficking of the WT cav-3 that properly traffics close to the plasma membrane.

Since this mutation is located within the CSD, a region important in the multimerization of caveolin monomers, one might expect a negative dominance of the mutated protein towards WT and an intracellular retention of both, yet this mutation appears to prevent interaction between the two isoforms, leaving the WT protein free to form homo-oligomers and to traffic normally.

In line with this view, patch-clamp data collected from MEF-KO expressing either the WT cav-3 alone or the WT/YTT together and co-transfected with the ion channels known to functionally interact with and be modulated by cav-3, hKv1.5, hHCN4 and hKir2.1, do not display differences either in current density or in kinetic properties, in both conditions. The lack of electrophysiological effects suggests that the independent trafficking of the WT cav-3 isoforms may sustain the formation of normally functioning caveolae.

The analysis of the T78K mutant, which is located at a bending point of the structure at the interface with the membrane, close to the CSD, reveals a very different effect. Our immunofluorescence and patch-clamp data clearly show that this mutant behaves as a dominant negative and indeed, besides accumulating in the perinuclear region, it retains the WT cav-3 in the same region. Moreover, all the ion currents analyzed in the heterozygous WT/T78K conditions displayed a significant reduction in density without alterations in the channels’ kinetic properties (see Figure 3, Figure 4 and Figure 5), indicating the ion channel trafficking impairments. This is fully compatible with the retention of caveolins within cells. The importance of this conserved residue (T78) was previously proven by our group with functional data demonstrating that its mutation into a methionine (T78M) has a higher “minor allele frequency” in patients affected by various cardiac pathologies such as atrial fibrillation, inappropriate sinus tachycardia and stillbirths than in the healthy population [4]. The T78M mutant, differently from the T78K, is able to reach the plasma membrane and to form normal caveolae, but the heterozygous expression of the T78M at the membrane level causes a gain in function in those ion channels physically interacting with cav-3, inducing a pro-arrhythmogenic cellular substrate [4]. Since T78K alterations induce a severe reduction in current densities, we investigated its potential arrhythmogenic effect in silico, using three different mathematical models of human atrial cardiomyocytes and a model of SAN cells. The in silico analysis confirmed that the recorded reductions of I_Kur_ and IK1 currents induced in two out of three models a repolarization failure not compatible with proper heart function, while the decrease in If induced a severe bradycardia (Figure 6). Since the W101C mutation also caused a smaller (29%) but significant decrease in I_Kur_, this was also modelled with the human atrial models, but no effects were found (Supplementary Figure S3). These data are fully compatible with the retention of caveolins and associated proteins (e.g., ion channels) within the cells. Nevertheless, these cardiac defects are not compatible with the so far normal ECG of the patients. 

In the literature, it has been shown that a patient with the T78K heterozygous mutation had only an isolated form of familiar hyperCKemia without any sign of cardiac involvement [16], even though both the WT and mutated cav-3 were retained within the cells; a condition that, as shown here, should severely affect the ion channel function and thus cardiac excitation. 

We hypothesized that the cellular context in which the effects of the cav-3 mutations are studied may be very important. For example, adult skeletal muscle does not express cav-1 while both neonatal and adult cardiomyocytes do [34] and it is possible that the presence of cav-1 may partially or completely rescue trafficking problems of specific cav-3 mutants. To verify our hypothesis, we repeated the electrophysiological analysis in cell lines endogenously expressing cav-1 (MEF-STO and CHO cells). In both cell lines, the WT and WT/T78K cav-3 conditions did not affect the functional properties of the studied cardiac ion channels (see Figure 7 and Supplementary Figure S3). To further validate this evidence, we created a MEF-STO line with a stable 78% reduction in cav-1 expression, and we repeated the electrophysiological analysis in the WT and WT/T78K conditions with the Kv1.5 channel. As in the MEF-KO cell, a 78% reduction in cav-1 was sufficient to decrease the Kv1.5 current density by 55%. 

In conclusion, our data provide evidence that the cellular system used for expressing mutated caveolins is important for the analysis of functional effects on cellular excitability. Moreover, we demonstrate for the first time that the higher susceptibility of skeletal muscle compared to the heart to develop pathological phenotypes may depend on the fact that this tissue expresses only cav-3. It must be emphasized that the transient expression obtained in heterologous cell models expressing cav-1 does not allow us to verify all the possible effects of the T78K mutation at the cardiac level, but did allow us to clearly show that the co-expression of cav-1 (as in the heart) is able to rescue trafficking of mutant cav-3 and partner proteins (e.g., ion channels) changing the membrane state at the cardiac versus skeletal level and this may protect the cardiac tissue from life-threatening events. 

### Study Limitations

We acknowledge that our study presents some limitations: first of all, we compared the effect of various cav-3 mutation in cell lines with a different genetic background (MEF-KO, MEF-STO and CHO cells). It is however important to keep in mind that in other muscle pathologies, the cardiac phenotype is not always manifested together with skeletal muscle phenotypes. For example, in Duchenne’s muscular dystrophy patients, the major and early phenotypes include only the skeletal muscle. With advancements in medical care, however, cardiac problems became manifest in a high proportion of Duchenne’s patients and constitute the second cause of death [35]. The use of different cell lines with different backgrounds may seem a limitation; if from one side this makes a direct comparison difficult, on the other side the lack of effect of the T78K mutation in various lines with different backgrounds justifies the central hypothesis of the importance of caveolin-1 expression as a general and widespread cardiac protective mechanism. This is even more evident when cav-1 has been significantly downregulated in MEF-STO cells in which the I_Kur_ current becomes “sensitive” to the expression of the T78K mutation (see Figure 8). 

We are convinced that other physiological models like those generated from patient-derived hiPSCs may be able to shed light on additional possible mechanisms that could increase these patients’ risk of arrhythmia. In this work, we offer proof of the possible role of cav-1 in mitigating the effects of specific cav-3 mutations linked to dystrophic phenotypes. It would also be important to test our “protective” hypothesis in this more physiological human cellular system. Although very interesting, the analysis of iPSC-derived cardiomyocyte is beyond the scope of this work.

## 4. Materials and Methods

### 4.1. Cell Culture, Transfection Procedure and Cell Imaging

Caveolin-free mouse embryonic fibroblasts from (3T3 MEF-KO CRL-2753™, ATCC, Manassas, VA, USA) were maintained in DMEM high glucose (Thermo Fisher, Waltham, MA, USA), supplemented with 2 mM L-Glutamine (Sigma-Aldrich^®^, Saint Louis, MO, USA), 10% FBS (Euroclone, Milan, Italy) and 1% Penicillin-Streptomycin (Pen-Strep, Sigma-Aldrich^®^). Immortalized Mouse embryonic fibroblasts (STO CRL-1503, ATCC) endogenously expressing cav-1 were maintained in DMEM high glucose (Thermo Fisher) supplemented with 4 mM L-Glutamine, 0.15% Sodium Bicarbonate (Sigma-Aldrich^®^), 10% FBS (Euroclone) and 1% pen-strep. Cells plated in 35 mm dishes were co-transfected with 1 µg of either pEGFP-N1 expressing WT or mutated cav-3, or with 0.5 µg of both, together with 1 µg of pCDNA 3.1 plasmids containing the ion channel sequences for hHCN4, hKv1.5, hKir2.1. We used Fugene HD Transfection Reagent (Promega, Madison, WI, USA) following manufacturer’s guidelines. 

Cav-3 sequences were subcloned into the pEGFP-N1 vector and mutagenized using the following primers:

∆YTT_For GGCGTGTGGAAGGTGAGCTTCACTGTCTCCAAGACT;
∆YTT_Rev AGTCTTGGAGACAGTGAAGCTCACCTTCCACACGCC;
T78K_For CCGTCTGTTGTCCAAGCTGCTGGGCGT;
T78K_Rev ACGCCCAGCAGCTTGGACAACAGACGG;
W101C_For TTCTGCCACATCTGCGCGGTGGTGCCATG;
W101C_Rev CATGGCACCACCGCGCAGATGTGGCAGAA.

For experiments requiring co-transfection of both WT and mutated cav-3, the plasmid containing the WT cav-3 was modified substituting the EGFP with the sequence encoding for the DsRed protein. 

To perform cell imaging, cells were directly seeded onto glass slides (Sarstedt, Nümbrecht, Germany) and transfected with 0.25 µg of both WT and mutated-cav-3 using Fugene HD Transfection Reagent, and 36–40 h post transfection the DsRed and EGFP signals were acquired using a Video confocal microscope (ViCO, Nikon, Tokyo, Japan). 

### 4.2. DNA Sequencing and Immunohistochemistry of Skeletal Muscle Biopsies

The sequencing analyses were carried out on peripheral blood mononuclear cells obtained from venous peripheral blood samples using the following primers.

For: TGTGGGCACCTACAGCTTTGAC; Rev: CACCTGGCTTTAGACCTCCTTC. 

Unfixed 5-μm-thick cryosections of skeletal muscle biopsies from a healthy control and the patient carrying the p.T78K sequence variant were incubated (1 h at room temperature, RT) with a primary anti-cav-3 mAb mouse, diluted 1:800 in PBS and /1% BSA (Transduction Laboratories, Lexington, KY, USA). After washing in PBS, sections were incubated for 1 h at RT with a biotinylated anti-mouse IgG (1:100, Amersham Biosciences, Little Chalfont, UK) and exposed for 30 min at RT to streptavidin fluorescein (1:250 Amersham Biosciences) in the dark. Sections were mounted with glycerol (87% diluted 2:1 in PBS) and observed under a Leica Diaplan microscope. Muscle biopsies were obtained after informed consent according to the guidelines of Gaslini’s hospital ethical committee. 

### 4.3. Cav-1 Silencing and WB Analysis

Cav-1 human shRNA Plasmid Kit (ORIGENE-TR314183) was used to obtain the knockdown of cav-1 in STO cells. We transfected 1 × 10^5^ cells with the pRS-HuSH plasmid containing either a scramble (SCR) or the shcav-1 sequence and a puromycin resistance cassette using the Fugene HD Transfection Reagent (Promega). Then, 40 h after transfection, cells were split and cultured in the presence of 2 μg/mL of puromycin into the medium. We kept cells under puromycin selection for 1 week and selected the growing clonal cells. After 10 passages, cells were solubilized in RIPA buffer with added protease inhibitors. Then, 30 µg of total proteins were loaded and run on pre-casted 8–12% gel (NuPage Thermo Fisher) and transferred onto PVDF membranes. The PVDF membrane was incubated overnight at 4 °C with anti-cav-1 (Abcam, Cambridge, UK; 1:1000 in TBS-tween) and anti-tubulin (1:2500 in TBS-Tween) antibodies. After a few washes in TBS, membranes were incubated with HRP-conjugated anti-rabbit IgG (for cav-1) and anti-mouse IgG (for tubulin) antibodies. The SuperSignal™ West Femto Maximum Sensitivity Substrate (Life Technologies, Carlsbad, CA, USA) was used for the chemiluminescence detection. 

### 4.4. Electrophysiology

Cells were superfused with a Tyrode’s solution containing 140 mM NaCl, 5.4 mM KCl, 1.8 mM CaCl_2_, 1 mM MgCl_2_, 5.5 mM D-glucose, 5 mM Hepes-NaOH; pH 7.4. HCN4 currents were recorded using the Tyrode’s solution to which 1 mM BaCl_2_, and 2 mM MnCl_2_ were added to inhibit background potassium and calcium conductance; pH 7.4. Patch-clamp pipettes were used with a resistance of 5–7 MΩ when filled with the intracellular-like solution containing the following: 130 mM KCl, 10 mM NaCl, 5 mM EGTA-KOH, 0.5 mM MgCl_2_, 2 mM ATP (Na-salt), 5 mM creatine phosphate, 0.1 mM GTP, 10 mM Hepes-KOH; pH 7.2.

hKv1.5 activation curves were obtained from tail currents at −40 mV after activating the current by 200 ms depolarizing steps in the range −60/+60 mV in 10 mV increments. Inactivation curves were obtained from tail currents at 50 mV after activating the channel with 10 s long depolarizing test steps in the range −60/50 mV. Holding potential (hp) was set at −80 mV. hHCN4 activation curves were obtained from tail currents at −125 mV preceded by test steps in the range −35/−125 mV in −10 mV steps (hp −30 mV). hKir2.1 current was analyzed as the Ba2+-sensitive component elicited by a voltage ramp in the range −100/+10 mV (hp −80 mV).

For current density analysis, current intensity at each potential was normalized to cell capacitance. Activation and inactivation curves were fitted to the Boltzmann equation: y = 1/(1 + exp((V − V1/2)/s)), where V is the test voltage, y the fractional activation, V1/2 the half-activation voltage, and s the inverse-slope factor (ISF). 

For each mutation current properties were analyzed recording both group of cells expressing either the WT or the WT/mutant cav-3, in three or more different transfections. 

Normality distribution was tested, and normally distributed data were compared by Student’s *t*-test, with *p* < 0.05 considered as significant. 

### 4.5. Computational Analysis

To verify the effects of the mutation in heterozygous conditions (Hetero WT/T78K and WT/W101C) in silico, different mathematical models of action potential (AP) were used. The following models were used: for human atrial cardiomyocytes, Grandi–Bers [22], Koivumaki [23], and the most recent Mazhar [24]; for SAN cells, the human Severi–DiFrancesco model [25] was used. The effects of the T78K mutations were simulated as experimentally reported: by reducing IKv1.5 current (I_Kur_) by 74.1%, Kir2.1 current (IK1) by 52.5%, and HCN4 current (I_f_) by 56.7% plus a +4.6 mV positive shift of HCN4 activation curve for the T78K heterozygous mutation. For the effects observed with the W101C we simulated a reduction in I_Kur_ by 29% plus a negative shift of both activation and inactivation of −5.4 mV and −3.7 mV, respectively. The atrial models were stimulated at 1 Hz for 500 beats for reaching the steady state and the SAN model was simulated for 100 s. To quantify the mutation effects on the AP we considered the action potential duration at 90% of repolarization (APD90) and the maximum diastolic potential (MDP); the results are reported in Supplementary Figure S2.

## Data Availability

Original data can be provided upon specific request to the corresponding authors.

## Supplementary Materials

The supporting information can be downloaded at: https://www.mdpi.com/article/10.3390/ijms25020980/s1.
