# Supplementary material for: Caveolin-3 and Caveolin-1 Interaction Decreases Channel Dysfunction Due to Caveolin-3 Mutations
Source: Int J Mol Sci. 2024 Jan 12;25(2):980. doi: 10.3390/ijms25020980 (PMC10816214; doi:10.3390/ijms25020980)
Supplement: Supplementary file 1 [file ijms-25-00980-s001.zip › ijms-2747857-supplementary.pdf]

Supplementary Figure 1

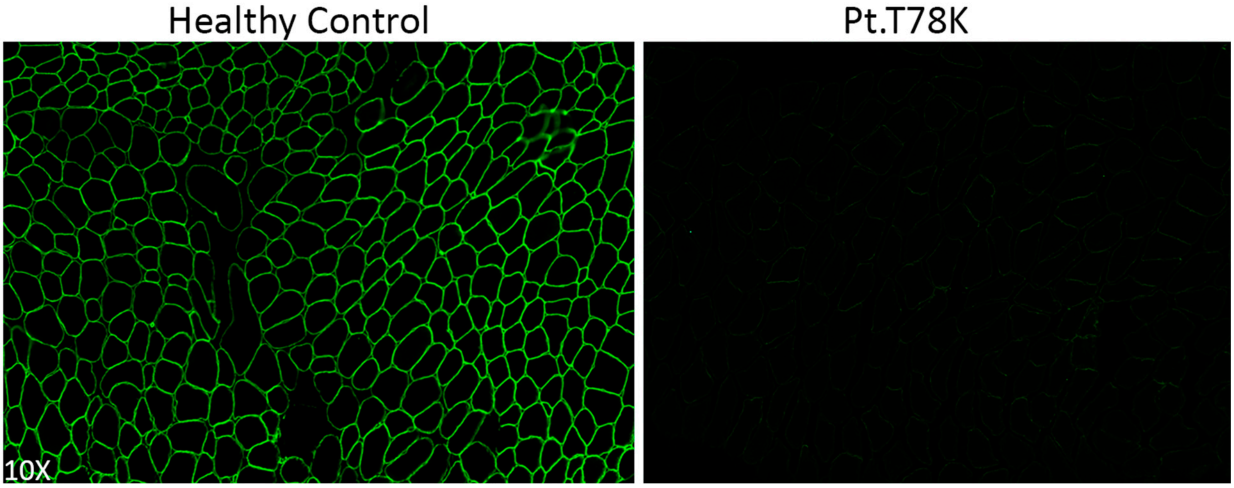

Figure S1. Immunohistochemistry of Muscle biopsies of T78K patient. Representative pictures of cav-3 expression in muscle biopsies. In control muscle, cav-3 displays a uniform pattern at the sarcolemma (left panel). CAV3 T78K mutation causes a severe decrease of cav-3 with an almost absent staining in the patient´s section (right panel). Final magnification, × 10.

Supplementary figure 2:

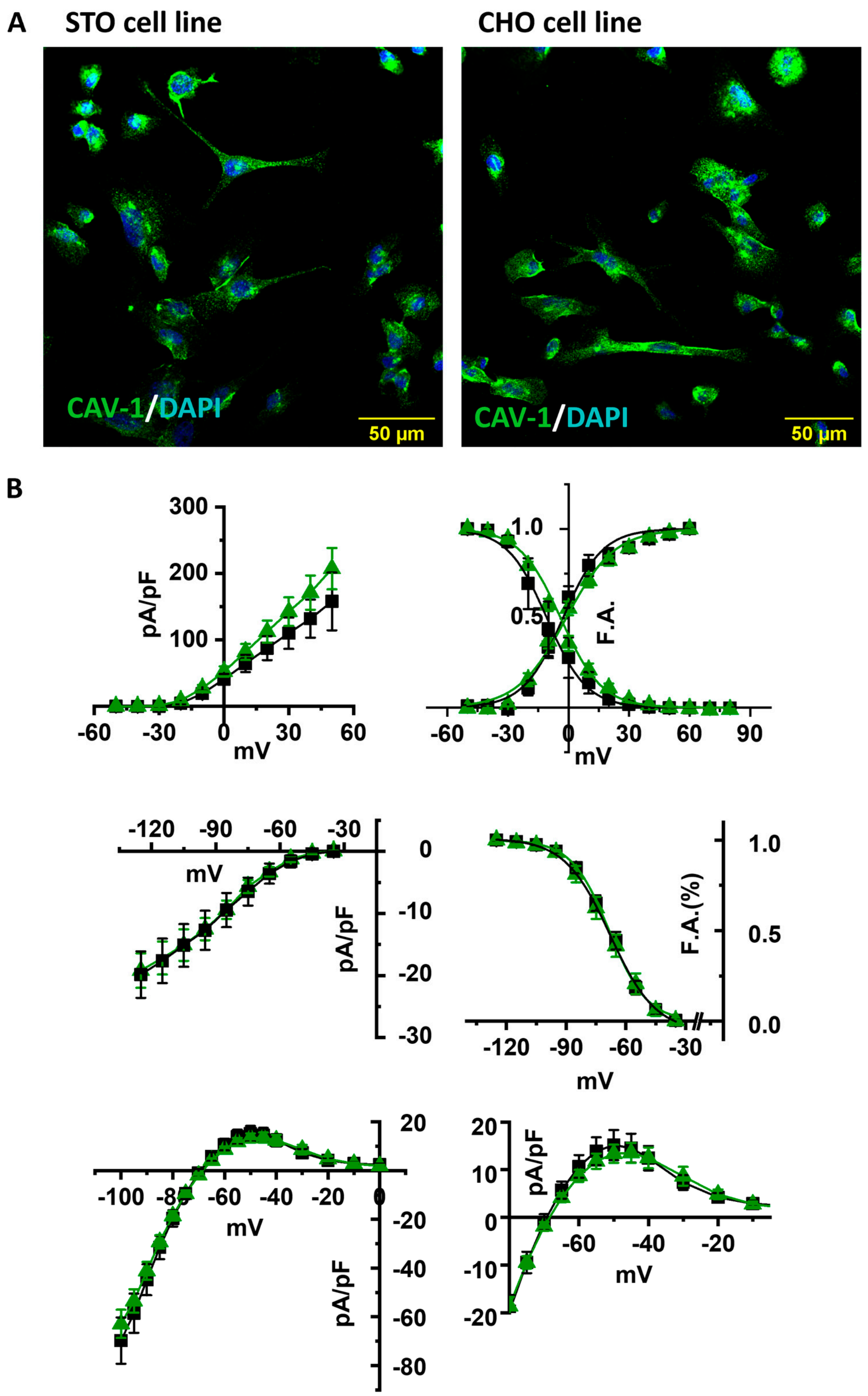

Figure S2. T78K mutation effect on cav-1 expressing cells. (A) Representative confocal images of STO-MEF left and CHO cells (right) stained with Abs displaying the endogenous expression of cav-1. (B) I-V relation of hKv1.5 current (top), hHCN4 current (middle) and hKir2.1 current recorded in transfected CHO cells with either WT Cav-3 or WT/T78K Cav-3. WT conditions are reported with black lines, and heterozygous conditions as green lines.

Supplementary figure 3.

A

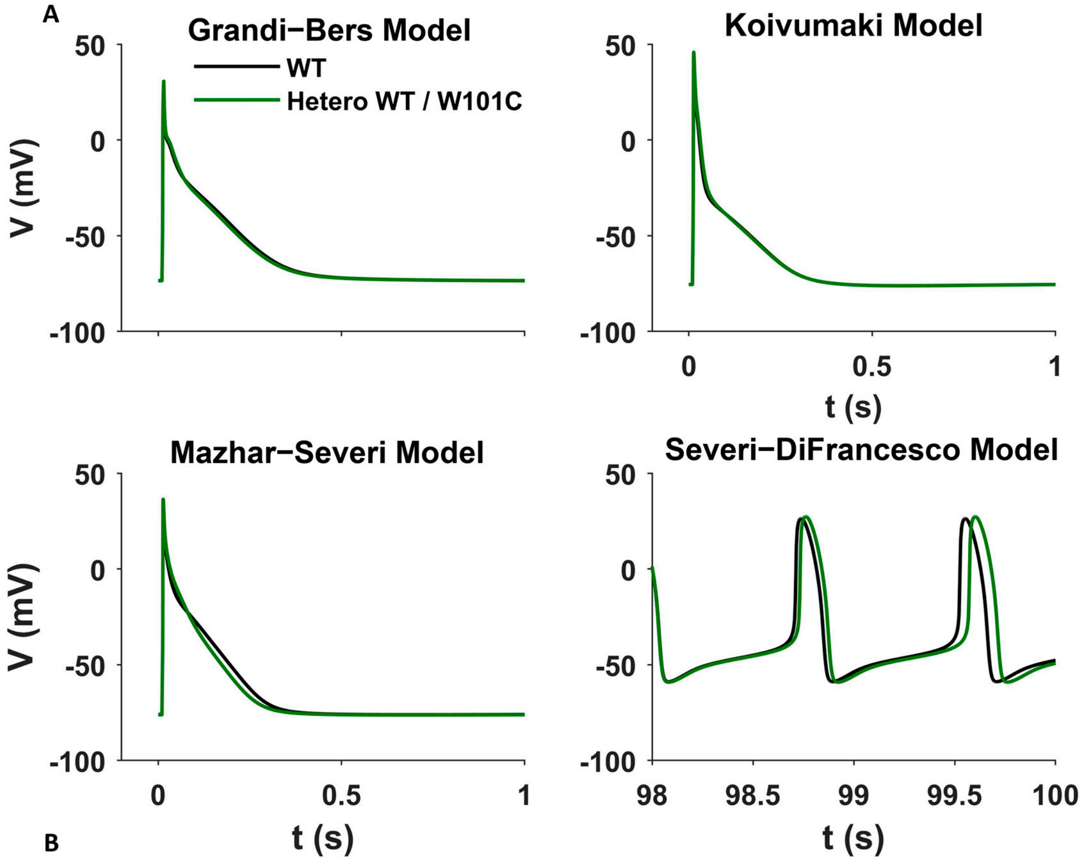

B

Models	APD$_{90}$/ CL (ms)	MDP (mV)	APD$_{90}$/ CL (ms)	MDP (mV)	ΔAPD90/ CL (ms)	ΔMDP (mV)
	Homo WT		Hetero WT/W101C			
Grandi−Bers	300	-73	290	-74	-10	-1
Koivumaki	231	-75	229	-75	-2	0
Mazhar−Severi	249	-76	225	-76	-24	0
Severi−DiFrancesco	813	-59	839	-59	+26	0

Figure S3. Computational Modelling of the W101C mutation effect. (A) Representative action potentials generated using the Courtmanche, Koivumaki and Grandi-Bers human atrial cell model showing the lack of any significant effect caused by the W101C cav-3 mutation. Representative action potentials generated using the Severi-DiFrancesco rabbit sinoatrial cell model. Black line, basal conditions (WT); green line, after insertion of the cav-3-W101C dependent alterations. (B) Summary table of the W101C effects on the different mathematical models analyzed.
